# Preparation of Polyurethane/Pluronic F127 Nanofibers Containing Peppermint Extract Loaded Gelatin Nanoparticles for Diabetic Wounds Healing: Characterization, In Vitro, and In Vivo Studies

**DOI:** 10.1155/2021/6646702

**Published:** 2021-05-15

**Authors:** Arash Almasian, Farhood Najafi, Mahdieh Eftekhari, Mohammad Reza Shams Ardekani, Mohammad Sharifzadeh, Mahnaz Khanavi

**Affiliations:** ^1^Department of Pharmacognosy, Faculty of Pharmacy, Tehran University of Medical Sciences, Tehran, Iran; ^2^Department of Resin and Additives, Institute for Color Science and Technology, Tehran, Iran; ^3^Department of Pharmacognosy, Faculty of Pharmacy, Kermanshah University of Medical Sciences, Tehran, Iran; ^4^Department of Toxicology, Faculty of Pharmacy, Tehran University of Medical Sciences, Tehran, Iran; ^5^Department of Pharmacognosy, Faculty of Pharmacy and Persian Medicine and Pharmacy Research Center, Tehran University of Medical Sciences, Tehran, Iran; ^6^Faculty of Land and Food Systems, University of British Columbia, Vancouver, Canada

## Abstract

Diabetic ulcer is regarded as one of the most prevalent chronic diseases. The healing of these ulcers enhances with the use of herbal extracts containing wound dressings with high antibacterial property and creating a nano-sized controlled release system. In this study, new peppermint extract was incorporated in the polyurethane- (PU-) based nanofibers for diabetic wound healing. The peppermint extract was used as an herbal antimicrobial and anti-inflammatory agent. The absorption ability of the wound dressing was enhanced by addition of F127 pluronic into the polymer matrix. The release of the extract was optimized by crosslinking the extract with gelatin nanoparticles (CGN) and their eventual incorporation into the nanofibers. The release of the extract was also controlled through direct addition of the extract into the PU matrix. The results showed that the release of extract from nanofibers was continued during 144 hours. The prepared wound dressing had a maximum absorption of 410.65% and an antibacterial property of 99.9% against *Staphylococcus aureus* and *Escherichia coli* bacteria. An in vivo study indicated on significant improving in wound healing after the use of the extract as an effective compound. On day 14, the average healing rate for samples covered by conventional gauze bandage, PU/F127, PU/F/15 (contained extract), and PU/F/15/10 (contained extract and CGN) prepared with different nanoparticle concentrations of 5 and 10 was 47.1 ± 0.2, 56.4 ± 0.4, 65.14 ± 0.2, and 90.55 ± 0.15%, respectively. Histopathological studies indicated that the wound treated with the extract containing nanofibers showed a considerable inflammation reduction at day 14. Additionally, this group showed more resemblance to normal skin with a thin epidermis presence of normal rete ridges and rejuvenation of skin appendages. Neovascularization and collagen deposition were higher in wounds treated with the extract containing nanofibrous wound dressing compared to the other groups.

## 1. Introduction

Recently, nano-sized drug delivery systems of herbal drugs have become an attractive topic for researchers due to their potential for enhancing activity as well as overcoming problems related to herbal medicines [1]. Polymeric nanoparticles, nanofibers, liposomes, and nano-emulsion, regarded as the novel drug delivery systems, have been widely used due to their unique advantages including bioavailability, high solubility, high pharmacological activity, nontoxicity, distribution, sustained delivery, and protection from physical and chemical degradation [2]. The novel drug delivery system is the method that an optimum amount of drug is administered to the patient which helps to minimize drug degradation and loss. However, it is important that the drug can be delivered to the patient continuously and in appropriate time. The efficacy of a drug is depended on the amount of drug delivered to the patient. For some drugs, an optimum concentration range exists and concentrations above or below this range produce no therapeutic effect. Also, the rate of delivering a drug to the patient is important. Very slow or very fast delivery progress influences on the treatment of severe diseases. In this regard, the control release of drugs is highly suggested [3].

To create controlled release systems, two strategies can be employed: (i) mixing the target drug with scaffold precursors during fabrication [4] and (ii) incorporating drugs into micro/nanocarriers [5]. These carriers should have superior properties, such as high encapsulation efficiency, keeping the drug preserved during storage, easy administration to the target site, and presentation of a controlled release rate [6]. Biodegradable polymers such as poly (lactic acid), poly (lactic acid-co-glycolic acid), poly (*ε*-caprolactone), and gelatin have been used as the carrier for producing micro- and nanospheres [7]. One method for creating a controlled release system is production of crosslinked structures with high swelling ability. Tannic acid, extracted from Chinese gall, is the gallic ester of d-glucose. Due to its multiple phenolic groups that can interact with biological macromolecules, tannic acid can play an excellent cross linker role [8]. Chitosan-gelatin sponge for absorbing surgical hemostatic materials was prepared by the use of tannic acid as the crosslinking agent [9].

Gelatin is a protein obtained through the partial hydrolysis of animal collagens, and it is known as a biocompatible and biodegradable polymer which has been widely used in pharmaceuticals, cosmetics, and food products [10]. Gelatin can also be easily crosslinked because of its intrinsic protein structure with the high number of different functional groups and used as a targeted drug delivery vehicle [11]. Crosslinked gelatin/nanoparticles composite coating on microarc oxidation film for corrosion and drug release are produced [12]. Also, gelatin nanoparticles as a swelling controlled delivery system for chloroquine phosphate are produced [13].

Diabetes mellitus is one of the most prevalent chronic diseases. The healing of these kinds of ulcers is too long due to poor perfusion, inflammation, and the presence of necrotic tissue [14]. The presence of moist, warm, and nutritious in wounds environment makes it an ideal place for the growth of microorganisms. In this regard, preparation of wound dressing with high antibacterial property is needed. Delivery of antimicrobial agents and a platelet-derived growth factor via biodegradable nanofibers for repair of diabetic infectious wounds was investigated [15].

The use of herbal extracts with several therapeutic effects and few side effects is highly suggested compared to chemical and synthetic drugs [16]. Many of these extracts accelerate wound healing due to their antimicrobial, anti-inflammatory, analgesic, and tissue regeneration properties. Curcumin-loaded poly (*ε*-caprolactone) nanofibers are prepared, and their diabetic wound healing is investigated [17]. In addition, polyurethane/carboxymethylcellulose nanofibers containing *Malva sylvestris* extract for healing diabetic wounds is prepared by our research team previously [14]. *Mentha piperita*, known as peppermint, has been widely used in folk remedies, food, cosmetics, and pharmaceutical industries as an antimicrobial agent. This plant also showed chemopreventive, antioxidant, and antimutagenic potential, renal actions, and antiallergenic effects [18]. It is used as an analgesic to treat headache. It has also antinematodal, antiviral, and antifungal properties [19]. Previously, peppermint essential oil was loaded into the polycaprolactone (PCL) electrospun fiber mats for wound healing [20].

The novelty of the present study can be summarized as follows: (i) the peppermint extract was not crosslinked with gelatin nanoparticles and its release behavior was not investigated, (ii) PU/F127 pluronic blend nanofibers was not prepared before, (iii) the peppermint extract was not loaded into a polymeric nanofibrous structure and its release behavior was not investigated, and (iv) the effect of peppermint extract on diabetic wound healing was not evaluated. In this study, peppermint extract was loaded in the crosslinked gelatin nanoparticles (CGN), and then, the prepared nanoparticles, beside to the extract, were incorporated on electrospun polyurethane PU/F127 nanofibers. The electrospinning method was used due to their attractive properties such as low cost, flexibility, integrability, relatively simple, and high efficacy [21].

## 2. Materials and Methods

### 2.1. Materials

Dried *Mentha piperita* L. (peppermint) was purchased from a local herbal drug market (Tehran, Iran) and was identified and deposited at the Herbarium of Pharmacy Faculty, Tehran University of Medical Sciences, Iran (PMP-1301). Gelatin, dimethylformamide (DMF), ethanol, and tannic acid were obtained from Merck. Pluronic F127 and polyurethane PU (*M*_*w*_ = 110,000) were purchased from Sigma-Aldrich and Cardio Tech., Japan, respectively.

### 2.2. Extract Preparation

Dried peppermint leaves (1000 g) were powdered and extracted by ethanol/distilled water (80/20) at ambient temperature using the maceration method in a constant time of 72 hours for three times by 6 liters solvent. The obtained extracts were combined, filtered, and dried under vacuum at 40°C.

### 2.3. Preparation of Crosslinked Gelatin Extract Nanoparticle

5 g gelatin powder was dissolved in 20 mL distilled water. 1 g extract was dissolved separately in 5 mL ethanol. The solutions were mixed together and stirred for 1 hour. Different amounts (0.3, 0.6, 0.9, and 1.2 g) of tannic acid were dissolved in 5 mL distilled water, and the solution was added dropwise to the mixture of gelatin and extract during 10 minutes. After that, the mixtures were heated to evaporate solvents. The obtained product was powdered using a dry ball mill. The powder was named as CGN (crosslinked gelatin nanoparticle).

### 2.4. Preparation of Nanofibers

PU solution was prepared by dissolving 2.1 g PU in 30 mL DMF. 0.21 g F127 was added to the solution to increase the hydrophilicity of final nanofibers. 15% w/w extract and different amounts of 5, 10, and 20% w/w CGN were added to the PU/F127 solution, and they were stirred for 30 minutes. The prepared solutions were electrospun under a high voltage range of 16–20 kV, and the distance between tip of needle and collector is 16 cm and feeding rate of 0.6 mL/h. The electrospinning apparatus was from Fanavaran Nano-Meghyas Co. (Iran). The electrospinning was performed at ambient pressure and temperature (25°C) and relative humidity of 40%. The obtained nanofibers were placed in a vacuum oven at room temperature for 48 hours to ensure the residual solvent is removed. The abbreviations of the names used in this study are presented in Table 1.

### 2.5. Characterization

#### 2.5.1. Physical and Chemical Characterization

The surface morphology of nanofibers was investigated using a field-emission scanning electron microscope (FE–SEM, Sigma, Zeiss Germany). Particle size and particle size distribution were determined by image analysis using an optical microscope (using the free Scion software) on a computer equipped with the Global Lab Image software, in the optical microscope, Jenaval–Carl Zeiss (Germany). The contact angle between nanofiber mats and the liquid phase was measured using the static digital method described in the standard D5725-97 (ASTM, 2003). To do this, an optical microscope (Olympus SZ-STU2) equipped with a digital camera (Olympus Camedia C-3040) was used. The FTIR spectrum of samples was examined by the FTIR spectroscopy (ThermoNicolet NEXUS 870 FTIR from Nicolet Instrument Corp., USA). The thermal degradation analysis (TGA) of the samples was performed on a TGA-PL thermoanalyzer from UK. In each case, a 5 mg sample was examined under N_2_ at a heating rate of 5°C/min from room temperature to 650°C.

#### 2.5.2. Assessment of Swelling Ratio

The swelling ratio (fluid absorption) of nanofibers was calculated as the following equation:

(1) $$\begin{array}{c} \text{Swelling ratio}\left(\%\right)=\frac{M-M_{d}}{M_{d}}*100, \end{array}$$

where *M* and *M*_*d*_ are the weights of the swollen samples and the dried samples in an oven at 40°C. Swollen sample was prepared by immersing 0.1 g of nanofibers in 50 mL of phosphate buffered saline (PBS, pH 7.4) at 35 ± 0.1°C for 24 hours.

#### 2.5.3. Assessment of Total Phenolic and Flavonoid Contents

In order to determine the total phenolic content, the Folin–Ciocalteu reagent was used [22]. To do this, 1 mL of the sample was mixed with 7.5 mL of distilled water and 0.3 mL of Folin–Ciocalteu's phenol (diluted 1 : 10). After 3 min, 1 mL of Na_2_CO_3_ solution (20%, w/v) was added to the mixture. The mixture was kept in a dark place for 1 hour, and then, its absorbance was measured at 760 nm using a single beam UV spectrophotometer. Results were expressed as gallic acid equivalents (mg of gallic acid (GAE)/mg dry weight extract).

The colorimetric method was used to evaluate the total flavonoid content [23]. In this method, different concentrations of a standard solution of catechin were added to 1 mL distilled water. Then, 75 ml of NaNO_2_ solution (5%) was added to the mixture. After 5 min, 75 mL of AlCl_3_ (10%) was added, and 6 min later, 500 mL of NaOH (1 N) was mixed to the solution. The mixture was diluted by adding 2.5 mL of distilled water, and its absorbance at the wavelength of 510 nm was determined using a spectrophotometer. Total flavonoid content was expressed as mg catechin equivalents (CE/g dry weight).

#### 2.5.4. Assessment of Release Behavior

The release behavior of effective compounds from nanofibers was investigated by immersing the nanofibers (0.1 g) in 40 mL of phosphate buffered saline (PBS, pH 7.4 at 37°C) in the time range of 0–160 h. The Folin method was used to investigate the amount of released compounds.

#### 2.5.5. Assessment of Water Vapor Transmission Rate

ASTM E96-00 standard was used to determine the water vapor transmission rate (WVTR). To do this, nanofibers were sealed over the circular opening of glass tubes filled with 10 ml distilled water. The diameter of tube was 13 mm. Then, it was placed in a chamber containing a saturated solution of ammonium sulfate at the constant temperature and humidity of 37°C and of 85%, respectively. The WVTR (g/m^2^·day) was determined as

(2) $$\begin{array}{c} WVTR=\frac{W_{i}-W_{t}}{A\times t}, \end{array}$$

where *A*, *T*, *W*_*i*_, and *W*_*t*_ designated the area of tube mouth (m^2^), the trial time, and the weights of trial before and after, respectively.

#### 2.5.6. Assessment of Antibacterial Activity

The colony counting method was used to evaluate the antibacterial activity of nanofibers against both Gram-positive *Staphylococcus aureus* (*S. aureus*, ATCC 6538) and Gram-negative *Escherichia coli* (*E. coli*, ATCC 8739) (33 m1). In this method, 60 mg of nanofibers was placed into 5 mL sterilized Luria–Bertani (LB) broth solution with the concentration of 1.5 × 10^5^ colony-forming units (CFU) of bacteria. The mixtures were cultured at 37°C in a shaking incubator for 12 hours. One hundred microliters of each of these cell solutions was seeded onto LB agar using a surface spread plate technique. The plates were incubated at 37°C for 24 hours. The numbers of bacterial colonies (CFU) were counted. Pure phosphate-buffered saline (PBS) was also tested as blank control.

(3) $$\begin{array}{c} \text{Antibacterial activity}\left(\%\right)=\left(\frac{B-T}{T}\right)*100, \end{array}$$

where *T* = cfu^*∗*^/ml of the test sample; cfu, concentration of colony of bacteria; *B* = blank sample, and cfu^*∗*^, colony-forming unit. The MICs of the extracts were determined by the agar dilution method.

#### 2.5.7. MTT Assay

The cell culture investigation was conducted by the protocol reported previously [24]. The human umbilical cord matrix (hUCM) cells were seeded at a seeding density of 1 × 10^6^ per well/1 mL medium in 96-well flat-bottom plates. After 24 h, the cultured cells were treated with different samples and free peppermint extract (dissolved in DMSO, the final DMSO concentration < 0.1%). Medium with 0.1% DMSO and control nanofibers were used as controls. Cytocompatibility of the wound dressings were characterized by using 3-4,5-dimethylthiazol-2-yl-2,5-diphenyltetrazolium bromide (MTT). Detecting of the formazan solution was conducted by a single beam UV spectrophotometer (CE-CIL CE2021) at 570 nm.

### 2.6. Preparation of Diabetic Rats and the Wound Healing Test

Male Wister weighing 150–200 g were used for diabetic experiments which were conducted according to Tehran University of Medical Sciences ethical guidelines for the care and use of laboratory animals (the ethical permission code is IR.TUMS.VCR.REC.1397.545). A single intraperitoneal injection of 70 mg/kg bodyweight of sterile streptozotocin (STZ) (Sigma, St Louis, MO, USA) in sodium citrate (0.1 mol/L, pH 4.5) was performed for inducing diabetes in rats. Animals were anesthetized by intraperitoneal injection of xylazine (13 mg/kg) and ketamine (66.7 mg/kg). The dorsal hair of rats was shaved, and 1.5 cm diameter full thickness wounds were created with a biopsy punch. The rats were randomly divided into four groups—each with five rats. The animal wounds covered by PU/F/15 and PU/F/15/10 nanofibers were classified as group 1 and group 2, respectively. The controls rats in groups 3 and 4 were treated with PU/F nanofibers and conventional gauze bandage, respectively. Wound dressings were changed every 4 days.

The healing rate was calculated with the following equation:

(4) $$\begin{array}{c} \text{Healing rate}\left(100\%\right)=\frac{\text{primitive area}-\text{nonhealing area}}{\text{primitive area}}*100. \end{array}$$

Wound observations were performed on rats that were sacrificed 3 days (*n* = 5), 7 days (*n* = 5), 14 days (*n* = 5), and 21 days (*n* = 5) after treatment. The wound dressings were changed every 4 days.

### 2.7. Histopathological Analysis

The animals from each group were euthanized 7, 14, and 21 days posttreatment, and the skin tissues were harvested and immediately fixed in the 10% neutral buffered formalin (PH = 7.26) for 48 h. The fixed tissue samples were then processed, embedded in paraffin, and sectioned to 5 mm thickness. Finally, the sections were stained with hematoxylin and eosin (H&E) and Masson's trichrome (MT). The histological slides were evaluated by the independent reviewer, using light microscopy (Olympus BX51; Olympus, Tokyo, Japan). Epithelialization, inflammatory cell infiltration, fibroplasia, and granulation tissue formation are assessed in different groups, comparatively.

### 2.8. Histomorphometry Analysis

On day 21, epithelialization was evaluated by a semiquantitative method. To do this, a 5-point scale, 0 (without new epithelialization), 1 (25%), 2 (50%), 3 (75%), and 4 (100%), was used. Evaluations were also scored in respect to angiogenesis according to the number of new vessels within the scar tissue, using a 5-point scale as follows: 0 (none), 1 (few), 2 (moderate), 3 (many), or 4 (considerably). Histomorphometric analysis was used to investigate neovascularization and collagen density. All experiments were analyzed using a computer software Image-Pro Plus® V.6 (Media Cybernetics, Inc., Silver Spring, USA).

### 2.9. Statistical Analysis

All results were compared using Kruskal–Wallis analysis. Results with *P* values of less than 0.05 were considered statistically significant. Statistical analyses were performed using the SPSS software, version 20.0 (SPSS, Inc., Chicago, USA).

## 3. Result and Discussion

### 3.1. Microscopic Analysis

**Figure 1:**
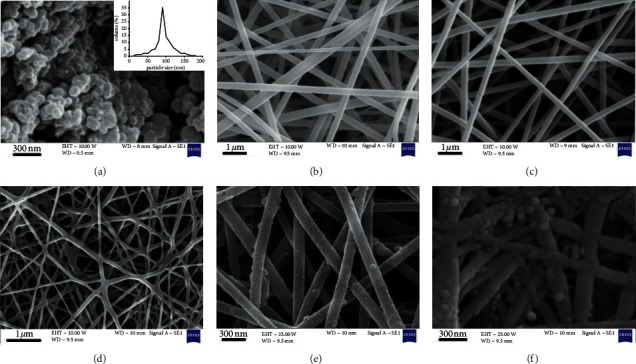
FE-SEM images of (a) CGN (inset image: pore size distribution curve of CGN), (b) PU, (c) PU/F, (d) PU/F/15, (e) PU/F/15/5, and (f) PU/F/15/10.

**Figure 2:**
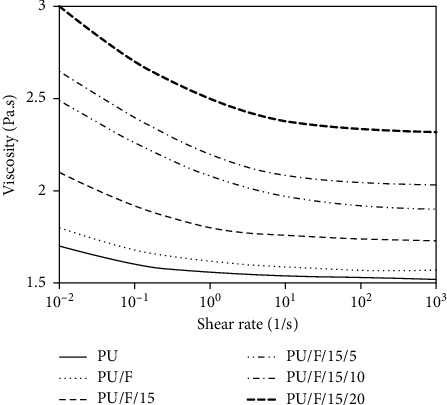
The changes of the viscosity as a function of the shear rate for different spinning solutions at 25°C.

The FE-SEM images of CGN, PU, PU/F, PU/F/15, PU/F/15/5, and PU/F/15/10 at different magnifications are shown in Figure 1. As can be seen, the shape of CGN was mostly spherical, and they had smooth surface. Particle size distribution curve of nanoparticles (inset of Figure 1(a)) showed a relatively narrow structure centered at 91 nm. PU nanofibers had smooth surface, and the average nanofibers diameter was 170.7 nm. After the addition of F127 into the PU matrix, the average nanofibers diameter increased to 176 nm. This can be due to the interaction of F127 with PU chains that resulted in increasing the spinning solution viscosity. The changes of the viscosity as a function of the shear rate for different spinning solutions at 25°C are shown in Figure 2. It was clear that the viscosity of solutions was increased after the addition of F127, extract, and CGN into the PU matrix. The increase of solution viscosity was more pronounced after the addition of CGN. This can be related to the stronger interaction of CGN with PU polymer due to higher number of functional groups in the gelatin structure. The shear thinning effect was observed for all samples. At low shear rates, the polymer chains absorb most of the applied energy, and they resist orienting in the flow direction resulting in high viscosity values. With increasing the shear rates, the structure of the polymer chains starts to break down and flows finally. The viscosity of PU, PU/F, PU/F/15, PU/F/15/5, PU/F/15/10, and PU/F/15/20 solutions at the shear rate of 10° was 1.56, 1.62, 1.8, 2.08, 2.2, and 2.5 Pa.s, respectively. According to Figure 1, for PU/F/15, some conglutinations especially at the touching points of nanofibers were seen. The presence of some nanoparticles on the surface of PU/F/15/5 and PU/F/15/10 nanofibers was detected. Nanofibers were not obtained when the CGN concentration was 20% w/w. This was related to the high viscosity value of spinning solution that resulted in unbalance condition of electrostatic repulsion, surface tension, and viscoelastic forces [25]. The number of nanoparticles on the nanofiber surface was increased when the CGN concentration in the spinning solution increased from 5% to 10% w/w. The average diameter of PU/F/15/5 nanofibers was 253.4 nm.

**Figure 3:**
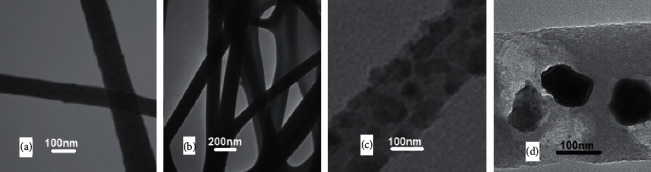
TEM images of (a) PU/F, (b) PU/F/15, and (c-d) PU/F/15/10.

TEM images of nanofibers are shown in Figure 3. It was clear that the F127 was dispersed into the PU matrix (Figure 3(a)). For PU/F/15 sample, a homogenous structure with some conglutinations was observed. It was found that the addition of extract in polymer matrix enhanced the interactions between PU and F127. Also, the presence of CGN in whole parts of PU/F/15/10 nanofibers was observed, indicating a good dispersion ability of CGN which can be related to the high functional group density of gelatin. The diameter of PU/F/15 and PU/F/15/10 obtained by TEM analysis was 210.5 and 278 nm, respectively.

### 3.2. FTIR Analysis

**Figure 4:**
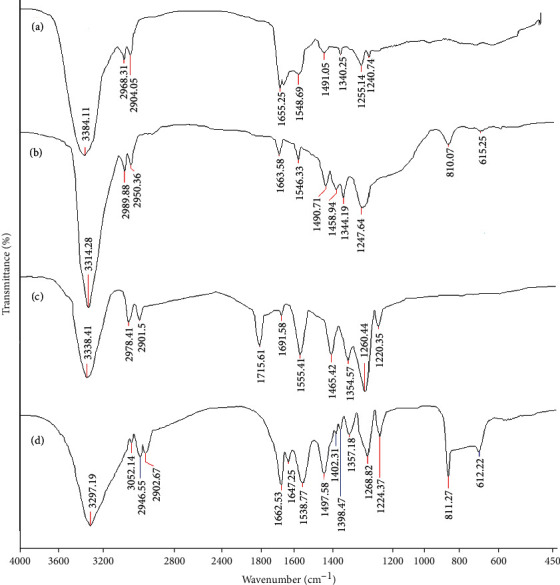
FTIR spectra of (a) gelatin, (b) CGN, (c) PU/F, and (d) PU/F/15/10.

The FTIR spectra of gelatin, CGN, PU/F, and PU/F/15/10 are presented in Figure 4. For gelatin, bands appeared at 1655 cm^−1^ and 1548 cm^−1^ were an indication of stretching vibration of the C=O bond (amid I) and coupling of bending vibration of the N-H bond and stretching vibrations of C-N bonds (amid II), respectively [26]. The band at 1655 cm^−1^, related to vibrations of amide I was split due to both random coil and *α*-helix conformation of gelatin [27]. The peak at 3384 cm^−1^ was related to the overlap of stretching vibrations of OH and NH groups. The asymmetric and symmetric stretching vibrations of methylene groups in the glycine and proline were reflected at 2968 cm^−1^ and 2904 cm^−1^, respectively [28]. The band at 1255 cm^−1^ was assigned to the overlapped stretching vibrations of C-N and N-H deformation from amide linkages. The peak observed at 1240 cm^−1^ was related to the asymmetric COC stretching vibrations [29].

After the crosslinking process, the bands at 3384 and 1255 cm^−1^ were shifted to the 3314 cm^−1^ and 1247 cm^−1^, respectively, which can be an indication of hydrogen bonding and protein conformation [30]. The peak appeared at 810 cm^−1^ attributed to the bending vibrations of C-H groups of phenyl rings. The peak at 615 cm^−1^ is assigned to CH out of plane bending vibrations of ethylene systems [31]. The appeared band at 1663 cm^−1^ was related to the stretching vibration of C=C in aromatic rings of phenolic compounds. The appeared band at 1490 cm^−1^ was attributed to the stretching vibration of C-C in aromatic rings of tannic acid [32].

For PU/F, the bands at 3338 cm^−1^ were related to the overlapped stretching vibration of OH and NH groups. The bands at 2977, 1715, and 1260 cm^−1^ were assigned to the stretching vibrations of C-H, C=O, and C-O in PU, respectively. The peak at 1220 cm^−1^ and 1555 cm^−1^ were attributed to the stretching vibration of C-O-C and bending vibration of the NH group, respectively.

For PU/F/15/10, the peaks appeared at 811 cm^−1^ and 612 cm^−1^ were related to the bending vibrations of C-H groups of phenyl rings and out of plane bending vibrations of CH groups of ethylene systems, respectively [31]. The appeared band at 1662 cm^−1^ was related to the stretching vibration of C=C in aromatic rings of phenolic compounds. Bands appeared at 1647 cm^−1^ and 1538 cm^−1^ were assigned to presence of CGN in the polymer matrix.

For this sample, the bands at 1224 cm^−1^ and 1268 cm^−1^ were intensified which indicated that the incorporated extract might have interacted with the ether groups of polymers through hydrogen bonds [33]. Also, the band, related to the vibrations of OH and NH groups, was shifted to lower wavenumbers (3297 cm^−1^). Shifting of bands towards lower wavenumbers was also detected for the bands at 1647 cm^−1^ and 1538 cm^−1^. Furthermore, the intensity of bands at 3297 cm^−1^ and 1628 cm^−1^ (related to the bending vibration of OH groups) was decrease compared to PU/F curve. These observations indicated on taking place interactions between extract ingredients' functional groups with hydroxyl and amino groups of polymers. Also, some interactions that occurred between CGN and PU resulted in increasing the solution viscosity. The presence of extract was confirmed by observing the peak at 3052 cm^−1^ which can be related to the stretching vibration of CH in aromatics. It was suggested that the extract and CGN play a crosslinking role in PU matrix.

### 3.3. TGA Analysis

**Figure 5:**
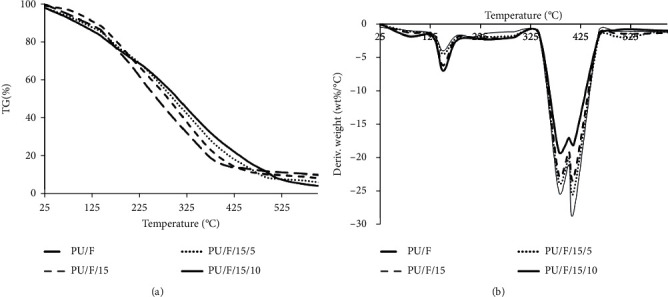
(a) TG and (b) DTG curves of different nanofibers.

The results of TG and DTG analyses, performed on different nanofibers, are shown in Figure 5. As can be seen, all curves had a similar 3-step structure including initial, main, and char decomposition. At the initial step (25.5–150°C), the weight loss for PU/F, PU/F/15, PU/F/15/5, and PU/F/15/10 was 14.34%, 12.7%, 15.78%, and 18.11%, respectively. The weight loss in this step was due to some physical damages, occurred in polymer chains with amorphous phases and the removal of the physically adsorbed water molecules [34]. The second step was the main thermal region in which the temperature ranged from 150°C to 400°C. In this step, the weight loss was related to the removal of chemisorbed water and degradation of polymer chains. It was clear that the thermal resistance was enhanced after the addition of CGN into the polymer matrix. This can be due to higher amounts of adsorbed water molecule (due to higher absorption capability of CGN containing nanofibers) and interaction of CGN, extract, and polymer together. Previously, it is reported that the presence of herbal extract in polymer matrix increases the hydrogen bonds of the components [35]. As mentioned in the FTIR section, the extract and CGN played a crosslinking role in the carrier polymer. Production of char occurred at the third step where the temperature was higher than 400°C. In this step, the release of chemisorbed water was continued and also carbon dioxide molecules were produced.

### 3.4. Mechanical Properties

**Figure 6:**
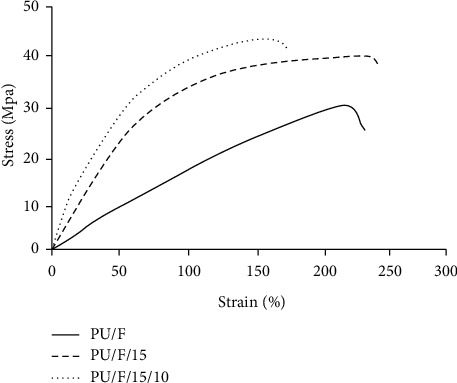
The stress strain curves of different nanofibers.

The stress strain curves of PU/F, PU/F/15, and PU/F15/10 samples are presented in Figure 6. It was found that the tensile strength and the elongation at break values of nanofibers enhanced due to the adhesive property and the plasticizer effect of extract [14, 36]. For PU/F/15/10 nanofibers, the tensile strength value was greater than the value for PU/F/15 sample, while the elongation at break value decreased compared to PU/F/15 nanofibers. As mentioned in the FTIR section, the crosslinked gelatin nanoparticles interacted with PU and herbal extract. Increasing the crosslinking degree resulted in decreasing the elongation property of nanofibers. Poor mechanical properties may generate some damage in the regenerated tissues at the change of wound dressing [37].

### 3.5. Antibacterial Properties

**Figure 7:**
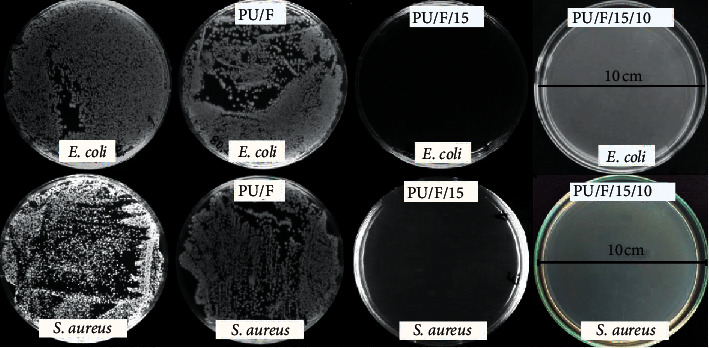
Antibacterial activity of different nanofibers.

Since chronic wounds remain open for long periods, they are highly exposed to the risk of bacterial infection. For these wounds, microorganisms can colonize and form a biofilm in the wound bed which develops high resistance against the antimicrobial agents and immune system [38]. The presence of a biofilm in the majority of chronic wounds is reported by researchers [39]. In this study, the results of the antibacterial test (Figure 7) showed that the PU/F nanofibers had no antibacterial property, while samples containing herbal extract had 99.9% antibacterial activity against both Gram-positive and Gram-negative bacteria. It is stated that the peppermint essential oil is made up of menthol, menthone, menthyl esters, and further monoterpene derivatives (pulegone, piperitone, and menthofuran) [40, 41]. Phenolic compounds that significantly contributed to the antibacterial activity of the herbal extract through the enzyme inhibition resulted from the reaction of phenolic compounds with sulfhydryl compounds or interactions with the protein [42]. Total phenolic content of the extract, determined by the Folin–Ciocalteu reagent, was 224.8 mg GAE/g. Total phenolic content is highly dependent on the solvent, used for extraction, the water content, and the time of extraction [43]. Phenolic constituents of peppermint extract were investigated previously, and quantity of compounds including caffeic acid, eriocitrin, luteolin-7-O-glucoside, naringenin-7-O-glucoside, hesperidin, isorhoifolin, rosmarinic acid, eriodictyol, and luteolin was determined [44]. Also, flavonoids synthesized by plants are effective against bacterial infections due to their ability to form complex with extracellular soluble proteins and with bacterial cell walls [45]. Total flavonoid content for peppermint extract was 200.47 mg CE/g. The MICs value against *S. aureus* and *E. coli* bacteria was 0.26 ± 0.01 and 0.29 ± 0.01 mg/ml, respectively. It was found that *S. aureus* was more sensitive to herbal extract than *E. coli* which can be related to variation in the structure of the cell wall [46].

### 3.6. Fluid Absorption and WVTR Tests

Ideal wound dressings should have superior properties including (i) absorbing excessive exudates, (ii) protecting the wound from bacterial infiltration, (iii) permeability of gaseous and fluid, (iv) removal ability without trauma, and (v) being nontoxic and nonallergenic [47]. Exudates facilitate the wound healing process; however, excessive levels of exudates result in tissue maceration and infections [48]. In this regard, regulation of moist wound is very important. The fluid absorption (%) values for PU, PU/F, PU/F/15, PU/F/15/5, and PU/F/15/10 were 2.77, 328.41, 316.73, 388.71, and 410.65%, respectively. Conventional PU wound dressings are nonabsorbent due to nonporous structure and hydrophobic nature of PU [49]. In this study, low absorption value of PU nanofibers can be attributed to the porous structure of nanofibers which enables the fluid molecules to penetrate into the voids existing among the nanofibers. It was clear that the addition of F127 into the PU matrix increased significantly the absorption value due to hydrophilic nature of F127. It was also discovered that the addition of extract into the nanofibers only slightly decreased the absorption percentage. This was related to the increased average nanofibers diameter. The presence of CNG in nanofibers enhanced the absorption percentage due to high absorption capacity of CGNs. The absorption value for CNGs prepared with 0.9% tannic acid was 494.26%. The hydrophilic nature of different nanofibers was investigated by the contact angle test. The water contact angle for PU, PU/F, PU/F/15, PU/F/15/5, and PU/F/15/10 nanofibers was 98.5, 25, 25.4, 20, and 19.2°, respectively. The results indicated that the addition of F127 significantly increased the hydrophilicity of nanofibers. Also, the presence of CGN on the nanofiber surface decreased the water contact angle values due to hydrophilic nature of CGN. Decreasing the water contact angle and enhancing the hydrophilicity property of PU nanofibers after the addition of herbal extract were reported by researchers [50].

The average WVTR values for PU, PU/F, PU/F/15, PU/F/15/5, and PU/F/15/10 were 357.24, 1880.63, 1854.05, 2095.24, and 2255.47 g/m^2^·day, respectively. Low WVTR value of PU nanofibers was related to the hydrophobic nature of polymer and low porosity of nanofibers. The porosity of PU, PU/F, PU/F/15, PU/F/15/5, and PU/F/15/10 samples was 59, 63.55, 69.4, 70.9, and 72.11%, respectively. Finally, it was found that the hydrophilicity of nanofibers played a more significant role than porosity in enhancing the WVTR values.

### 3.7. Release Behavior of Samples

**Figure 8:**
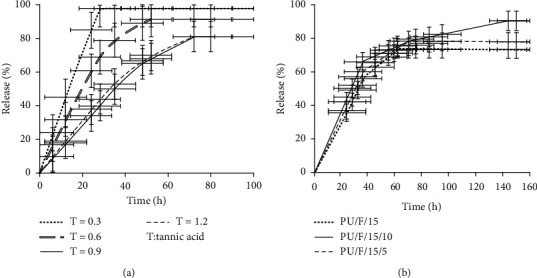
Release behavior of herbal compounds from (a) CGN and (b) different nanofibers.

The release behavior of samples was investigated, and the results are shown in Figures 8(a) and 8(b). In this study, beside to CGNs, the extract was mixed with polymer matrix, and its release behavior was investigated. In such systems, the pore size, crosslinking density of scaffold, and additives along with scaffold degradation could influence on the drug release rate. In this regard, at first, the release behavior of CGN was investigated, and the results are shown in Figure 8(a). As can be seen, CGN showed a gradual release up to 81.2% of the total amount of loaded extract during 72 h, and the apparent loss was related to covalent binding of herbal compounds to the polymer matrix. Such a result is reported by researchers [51]. From the figure, it was obvious that the released amount of extract was dependent on the tannic acid concentration in crosslinking procedure. With increasing the content of tannic acid from 0.3% to 1.2%, the amount of released extract from nanoparticle decreased from 97.8% to 81.21%. The solubility (%) of CGN prepared with different amounts of 0.3, 0.6, 0.9, and 1.2% tannic acid was determined as 21.14, 10.75, 0.1, and 0.09%, respectively. It was concluded that the gelatin structure was fully crosslinked when the amount of tannic acid was higher than 0.9%. The result showed that the increase of CGN crosslinking degree resulted in increasing the releasing time up to 72 h. The equilibrium time point of CGN prepared with 0.3, 0.6, 0.9, and 1.2% tannic acid was 28, 52, 72, and 72 h, respectively. In this regard, the amount of 0.9% tannic acid was selected as the optimum.  Figure 8(b) shows the release behavior of different nanofibers. It was obvious that the release profile of PU/F/15 presented a three-step structure. At the first step (0–36 h), a quick release of extract was seen, and the amount of released extract was 56.02%. The entrapped extract on the surface of nanofibers is responsible for the intense slop of the release profile during the first 36 h. The incorporation of extract on the nanofiber surface is attributed to the limited physical interactions between the extract and the polymer matrix in the electrospinning process [22]. However, the presence of F127 with the large number of ethylene oxide groups increased the hydrophilic property of nanofibers. This was resulted in increasing the extract diffusion in the polymer matrix, and the release of extract was continued in longer times. The amount of released extract in the second step was 17.15%, and the equilibrium point was 62 h. For PU/F/15/10 sample, the amount of released extract showed a 17.8% increase at the time of 36 h. This increase was attributed to the released herbal compound from nanoparticles distributed on the surface of nanofibers. After 62 h, the releasing of herbal compounds was continued for the next 82 h, and the equilibrium point was achieved at 144 h. At the initial hours, the detected extract in the solution was attributed to the mixed extract with polymer blend. After that, the swelling of nanofibers caused penetration of water molecules into the inner layers of polymer and nanoparticles. Thus, the swelling of nanoparticle was begun at longer times that resulted in gradual release of extract during 62–144 h. The total release amount of peppermint extract for PU/F/15/10 was 17.42% higher than PU/F/15. It is reported that the drug release from gelatin nanoparticles can be occurred according to three mechanisms including desorption, diffusion, and biodegradation of nanoparticles [52]. It was suggested that desorption of surface-bound/adsorbed and diffusion through the carrier matrix were the main mechanisms in releasing herbal compounds from nanoparticles. Fully crosslinking of gelatin nanoparticles caused to emerge a controlled release behavior of nanofibers. Similar behavior was observed for PU/F/15/5. The amount of released extract at the time of 36 h was 60.1%, and the equilibrium time point was 95 h.

### 3.8. MTT Assay

**Figure 9:**
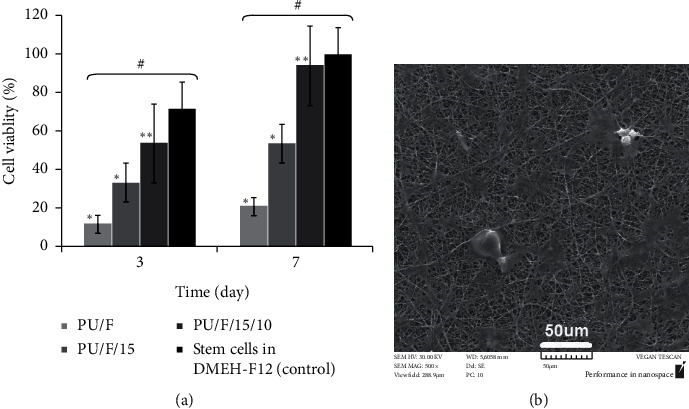
(a) The viability of wound dressings after 3 and 7 days, Data are expressed as mean ± SD from three individual experiments.*∗*, *p* < 0.05, ∗∗, *p* < 0.01, and ^#^*p* < 0.001. (b) SEM images of hUCM cells after 7 days seeded on PU/F/15/10 nanofibers.

The result of the MTT test for PU/F and PU/F/15/10 at days 3 and 7 is shown in Figure 9. It was found that the absorbance values at the day 7 showed an increase of 160% and 176.6% for PU/F/15 and PU/F/15/10 compared to the absorbance values of day 3, respectively. The number of grown cells on PU/F/15/10 nanofibers was much higher than PU/F nanofibers. Also, the absorption values of PU/F/15 and PU/F/15/10 samples were 75% and 94.64% of absorption values of control samples, respectively, indicating on the nontoxic nature of the prepared wound dressing. This can be due to natural and organic nature of herbal extract. As mentioned in contact angle and WVTR analyses, the hydrophilicity of nanofibers increased after the addition of extract. Increasing the moist and porosity of the scaffold resulted in enhancing the cell compatibility and grown cells number. Furthermore, the surface of extract containing samples showed high antibacterial property, which makes these nanofibers a suitable place for cell growing. The presence of hUCM cells on the wound dressings after 7 days was investigated by microscopic analysis, and the result is shown in Figure 9(b). High accumulation of stem cells on nanofibers was an indication of nontoxic nature of nanofibers and the effectivity of this scaffold in cell proliferation.

### 3.9. Wound Healing and Histological Examination

**Figure 10:**
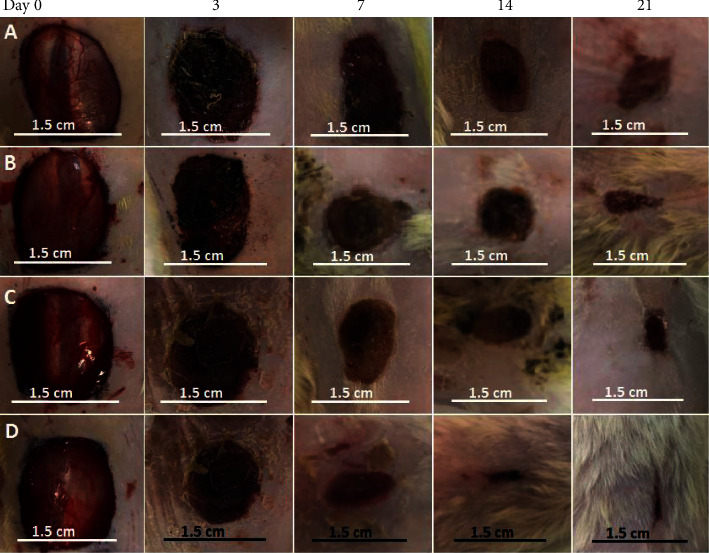
Representative wounds on an animal on days 0, 3, 7, 14, and 21 after treatment for (a) conventional gauze bandage, (b) PU/F, (c) PU/F/15, and (d) PU/F/15/10.

**Figure 11:**
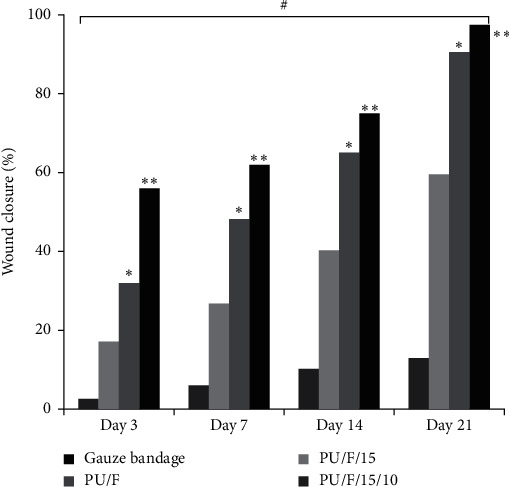
The effect of different wound dressings on healing of diabetic wounds. *∗*, *p* < 0.05, ∗∗, *p* < 0.01, and ^#^*p* < 0.001.

Wound healing mainly involves three phases including inflammation, proliferation, and wound closure and remodeling. In diabetic patients, impaired wound healing resulted from abnormal inflammatory response and insufficient fibroblast proliferation. A representative wound on an animal in each group and the wound healing percentages of diabetic rats on days 0, 3, 7, 14, and 21 after treatment are shown in Figures 10 and 11, respectively. As can be seen, there were no significant changes in wound area for sample covered with gauze bandage on day 3. This was related to the lack of moisture on the surface of the wound caused to generate some damage in the regenerated tissues at the change of gauze bandage. For the sample covered with PU/F nanofibers, better healing (higher wound closure percentage) was observed due to the higher absorption ability of PU/F wound dressing in comparison with gauze bandage that resulted in taking up wound exudates and providing a moist wound healing environment [53]. Also, relatively hindering property of the prepared scaffold from the penetration of bacteria into the wound surface can be another reason for higher efficiency of PU/F compared to gauze bandage. Delaying of the wound healing rate in wounds covered with gauze bandage and PU/F compared to the other groups was attributed to the existence of bacteria in wounds or because of their histopathological lesions [54]. The wound healing percentages of diabetic rats on day 3, treated with gauze bandage, PU/F, PU/F/15, and PU/F/15/10, were 2.55 ± 0.1, 5.84 ± 0.2, 10.11 ± 0.1, and 12.75 ± 0.25%, respectively. The results showed that the wound contraction process occurred rapidly for wound treated with extract containing wound dressings compared to the other dressings. This can be related to reducing the inflammation phase time and acceleration in entry into the proliferation phase [55]. The wounds treated with PU/F/15 and PU/F/15/10 showed a significant closure on day 7 compared to other wounds. The wound closure for these samples was 40.22 ± 0.5 and 59.47 ± 0.2%, respectively. As can be seen, the healing percentage for PU/F/15/10 treated wound was much higher than PU/F/15. The results of wound healing were in agreement with the results of the release test. The release equilibrium point of PU/F/15 was 59 h, and after that, the amount of released compound was close to zero. Since the wound dressings were changed after 4 days, the possibility of growing bacteria on the wound surface increased and the effective herbal drugs was not in contact with wound.

The wound healing percentages for samples covered with PU/F/15 and PU/F/15/10 were 65.14 ± 0.2 and 90.55 ± 0.15% on day 14, respectively. These values enhanced to 75 ± 0.22 and 97.5 ± 0.1% on day 21, respectively. It was clear that the wound healing for PU/F/15/10 treated wound proceeded faster than PU/F/15 treated wound. This can be attributed to the controlled release of herbal extract that resulted in contact of extract continually to wound surface and promote wound healing. Also, antioxidant and antibacterial properties of peppermint can facilitate the wound contraction and wound healing [49].

**Figure 12:**
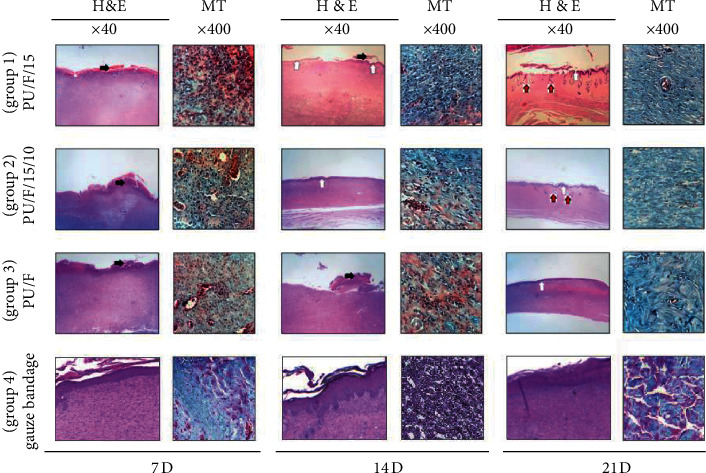
H&E and MT stained microscopic sections of healed incisions in treatment groups, Black thick arrows, crusty scab; white thick arrow, epidermal layer; red thick arrows, rejuvenation of skin appendages.

Histopathological images on days, 7, 14, and 21 are shown in Figure 12. For PU/F/15 and PU/F/15/10 covered samples, large number of inflammation cells was observed on day 7, while it was too low for PU/F-treated wound. Inflammation occurs at the initial step of the wound healing process, and uncontrollable inflammatory response is associated with unhealed diabetic ulcer [56]. Also, the presence of necrosis was detected for all samples. For sample covered with PU/F/15/10 nanofibers, regenerative responses and tissue granulation were observed on day 7. The granulation tissue is an important indicator for evaluating wound healing [57]. For PU/F/15 treated wound, the crusty scab covered the wound area, and the inflammation was also evident in the defect on day 14. At day 14, formation of fibroblast cell, collagen fibers, and connective fibrils were observed for PU/F/15/10-treated wound. It was reported that most of herbal drugs have anti-inflammatory property which accelerates collagen fiber development and epithelium regeneration [58]. Higher production of the collagen matrix correlates with increased scar formation. As can be seen, for PU/F/15/10 treated wound, the inflammation was significantly alleviated, and regeneration of the skin was more pronounced on day 14 compared to the other samples. For this sample, the collagen turnover was higher than PU/F/15 which was attributed to the higher released amounts of extract. The higher collagen turnover results in the higher granulation rate of tissue components [59]. A thick layer of reepithelialization and small blood vessels intermingled with fibroblastic cells were seen for PU/F/15 and PU/F/15/10-treated wounds on day 21. For PU/F/15/10, the thickness of reepithelialized layer and the number of blood vessels were higher than PU/F/15. The presence of menthol with various biological properties, such as antimicrobial, anticancer, and anti-inflammatory agent, could reduce the inflammation and facilitate of nerve regeneration [60, 61].

### 3.10. Histomorphometric Analysis

The histomorphometric analysis for PU/F/15 (group 1), PU/F/15/10 (group 2), and PU/F (Control, C) was performed at 7, 14, and 21 days after skin injury, and the results are presented in Tables 2 and 3. As can be seen, reepithelialization in the group treated with extract containing nanofibers (groups 1 and 2) was better than other treatment groups at day 14. The wound healing process is heavily dependent on collagen synthesis. Therefore, to further investigate the effect of different treatments on wound healing, sections of animal skin tissues were stained with MT staining (Figure 11). This staining was used to recognize the progress of collagen synthesis during GT formation and matrix remodeling. Collagen fibers were stained blue-green in the MT staining method, in which the intensity of this color corresponds to the relative amount of deposited total collagen and reflects the advancement of collagen synthesis and remodeling. The results indicated that the groups 1 and 2 had the greatest collagen synthesis. In contrast, the rate of collagen fiber synthesis and deposition in the wound were the lowest in group C. Reducing the inflammation phase time, accelerating in entering to the proliferation phase, tissue granulation, and wound contraction and higher collagen production were assigned to healing mechanism.

## 4. Conclusion

In this study, hydrophilic polyurethane-based wound dressings containing peppermint extract were prepared by electrospinning technique. The extract release was controlled by the addition of crosslinked extract-gelatin nanoparticles with addition to direct use of extract in the polymer matrix. The equilibrium releasing time and the maximum buffer absorption were 144 h and 410.65%, respectively. FTIR results indicated on generating of hydrogen bonds between the extract, CGN, and carrier polymers. Antibacterial and control release properties of the extract containing wound dressings were the reason of the fast and acceptable wound healing process. The wound closure percentage for PU/F/15 and PU/F/15/10 samples was 65.14 ± 0.2 and 90.55 ± 0.15%, respectively, on day 14. The result of MTT analysis indicated the nontoxic nature of the wound dressing. Histopathological analysis revealed that the use of extract containing wound dressings reduced the inflammation phase time and caused to accelerate entry into the proliferation phase. Also, regenerative responses, tissue granulation, and wound contraction proceeded faster than control. Formation of fibroblast cell, collagen fibers, and connective fibrils were more observable in extract containing scaffold treated wound. Higher production of the collagen matrix correlates with increased scar formation. The higher collagen turnover results in the higher granulation rate of tissue components. A thick layer of reepithelialization and small blood vessels intermingled with fibroblastic cells were seen. Further studies related to advanced investigations such as cell migration, hemolysis, monitoring blood serum cytokine levels, and human cytokine synthesis inhibitory factor can be evaluated to understand the mechanism of wound healing in a better and accurate way. In conclusion, the prepared nanofibers showed potent wound healing activity for diabetic ulcers. Also, the prepared scaffolds have potential for applications in antibiotic-free bacterial infection treatment as wound healing materials.

## Figures and Tables

**Table 1:** The abbreviations of the names.

Name	Polyurethane	Polyurethane/Pluronic F127	Polyurethane/Pluronic F127/Dried peppermint extract (containing 15% w/w extract respect to PU)	Crosslinked gelatin nanoparticles	Polyurethane/Pluronic F127/Dried peppermint extract (15% w/w)/Crosslinked gelatin nanoparticles (containing 10% w/w CGN) containing 0.9 g tannic acid and 1 g peppermint extract
Abbreviation	PU	PU/F	PU/F/15	CGN	PU/F/15/10

**Table 2:** Histomorphometric analysis of different experimental groups.

Group	Angiogenesis	Epitheliogenesis (score)
1 (PU/F/15)	1 (7 d)	0 (7 d)
	1 (14 d)	1 (14 d)
	2 (21 d)	4 (21 d)

2 (PU/F/15/10)	1 (7 d)	1 (7 d)
	2 (14 d)	3 (14 d)
	3 (21 d)	4 (21 d)

3 (PU/F)	1 (7 d)	0 (7 d)
	1 (14 d)	0 (14 d)
	2 (21 d)	3 (21 d)

4 (gauze bandage)	0 (7 d)	0 (7 d)
	1 (14 d)	0 (14 d)
	2 (21 d)	2 (21 d)

**Table 3:** Histomorphometric analysis of different inflammatory cells and collagen deposition (%).

Group	Inflammatory cells/3HPF	Collagen deposition (%)
1 (PU/F/15)	158 (7 d)	34.3 ± 3.0 (7 d)*∗*
	101 (14 d)	55 ± 5.5 (14 d)∗∗
	42 (21 d)	80.3 ± 3.5 (21 d)

2 (PU/F/15/10)	132 (7 d)	39.6 ± 7.5 (7 d)∗∗
	83 (14 d)	64.6 ± 5.03 (14 d)∗∗
	23 (21 d)	88.6 ± 3.7 (21 d)

3 (PU/F)	175 (7 d)	21.0 ± 5.0 (7 d)
	136 (14 d)	31 ± 6.2 (14 d)
	79 (21 d)	50.0 ± 4.3 (21 d)

4 (gauze bandage)	179 (7 d)	17.2 ± 1.5 (7 d)
	142 (14 d)	25.5 ± 4.1 (14 d)
	80 (21 d)	40.8 ± 2.8 (21 d)

∗, ∗∗ values indicate the treatment group versus group C. *∗*, *P* < 0.05, ∗∗, *P* < 0.01.

## Data Availability

The data used to support the findings of this study are included within the article.
